# Shikonin Alleviates Endothelial Cell Injury Induced by ox-LDL via AMPK/Nrf2/HO-1 Signaling Pathway

**DOI:** 10.1155/2021/5881321

**Published:** 2021-12-06

**Authors:** Shuang Liu, Wen Yan, Yanbing Hu, Huiying Wu

**Affiliations:** ^1^Department of Ultrasound in the Second Hospital of Jilin University, Changchun, China; ^2^Department of Anesthesiology, The Second Hospital of Jilin University, Changchun, China

## Abstract

The present study aimed to explore the effects of shikonin (SKN) on the damage of human venous endothelial cells (HUVECs) induced by ox-LDL and the underlying molecular mechanism. The HUVECs were randomly divided into six groups: control, ox-LDL, SKN + ox-LDL, SKN + ox-LDL + compound C, SKN + ox-LDL + si-Nrf2, and SKN + ox-LDL + si-HO-1. The MTT method was used to detect cell viability, flow cytometry was used to detect cell apoptosis and reactive oxygen species (ROS) levels, and Western blot was used to detect protein levels. Compared to the control group, the cell viability of the ox-LDL group decreased, the apoptosis rate increased, the level of cleaved caspase-3 was upregulated, and the level of Bcl-2 protein was downregulated. The level of TNF-*α*, IL-1*β*, IL-6, vascular cell adhesion molecule-1 (VCAM1), intercellular adhesion molecule-1 (ICAM1), and E-selectin (E-sel) was increased, ROS levels increased, and superoxide dismutase (SOD) level decreased. Moreover, the protein levels of p-AMPK, Nrf2, and HO-1 were decreased. Compared to the ox-LDL group, SKN treatment improves cell viability, alleviates cell apoptosis and oxidative stress injury, and upregulates the protein levels of p-AMPK, Nrf2, and HO-1. Compound C, si-Nrf2, and si-HO-1 administration inhibits the AMPK/Nrf2/HO-1 signaling pathway, increases ROS generation, and inhibits the antagonistic effect of SKN on ox-LDL-induced HUVECs damage. In summary, SKN suppressed ox-LDL-induced ROS production and improved cell viability and cell apoptosis via the AMPK/Nrf2/HO-1 pathway.

## 1. Introduction

Atherosclerosis (AS) is a common clinical cardiovascular disease that affects coronary and carotid arteries, and cardiovascular and cerebrovascular adverse events caused by AS have become the primary cause of death in humans today [1]. A large number of studies have shown that many risk factors for AS (such as smoking, high blood pressure, diabetes, lipid metabolism disorders, hemodynamic changes, hypoxia, and infection) can cause inflammation and oxidative stress and lead to endothelial cell damage and dysfunction. In addition, inflammation, oxidative stress, and vascular endothelial damage are only a few significant features of AS [2].

Vascular endothelium is a major barrier for the maintenance of normal structure and function of the vasculature, and endothelial cell damage is the initial process of AS [3]. Studies have shown that endothelial cell damage causes deposition of low-density lipoprotein cholesterol (LDL-C) in large amounts under the inner membrane. After LDL-C is oxidized and modified to ox-LDL, it can be engulfed by macrophages to form foam cells that cause the occurrence and development of AS, effectuate abnormal vasomotor and contraction functions, and trigger thrombosis and cardiovascular events. Moreover, under the stimulation of oxidative stress factors, such as hydrogen peroxide, low-density lipoprotein, and proinflammatory factors, abundant active molecules, such as reactive oxygen species (ROS), produced in the body can disrupt the oxidant-antioxidant balance [4, 5]. Oxidative stress overactivates the endothelial cells, reducing the repair function and damaging the endothelial cells [6, 7]. Therefore, reducing the oxidative damage of oxidized low-density lipoprotein (ox-LDL) to endothelial cells is an effective way to prevent and treat AS.

The pathological basis of coronary heart disease is AS, and its occurrence is closely related to inflammatory factors. Studies have shown that interleukin-1*β* (IL-1*β*), IL-6, and tumor necrosis factor-alpha (TNF-*α*) are classic proinflammatory factors that participate in proinflammatory reactions, damage vascular endothelium, causing endothelial cell dysfunction, accelerate lipid deposition and matrix protein dissolution, reduce plaque stability, and promote the formation of atherosclerotic plaques [8].

Adhesion molecules, such as intercellular adhesion molecule-1 (ICAM1), vascular adhesion molecule-1 (VCAM1), and E-selectin (E-sel), are the initiating factors that play a major role in the early stage of atherosclerotic inflammation. These molecules are cytokine-inducible members of the immunoglobulin gene superfamily and expressed at a low level or not expressed on resting endothelial cells. However, the expression of these molecules is rapidly upregulated under the stimulation of ox-LDL and other inflammatory factors and in arterial endothelial cells of atherosclerotic lesions [9]. Thus, it is speculated that E-sel, VCAM1, and ICAM1 are the key molecules involved in the pathogenesis of AS inflammation. They facilitate the rolling of molecules on the surface of endothelium cells and cooperate to promote the adhesion of monocytes and then migrate to endothelial cells. A previous study has shown that the expression of E-sel, ICAM1, and VCAM1 is significantly increased in atherosclerotic plaques [8].

Nuclear factor E2-related factor 2 (Nrf2) is a key transcription factor in the antioxidant response. Under physiological conditions, Nrf2 and Kelch-like ECH-associated protein 1 (Keap1) form a complex. In the event of oxidative stress in the body, phosphorylation of a series of protein kinases causes Nrf2 to decouple from Keap1 and transfer to the nucleus to bind to the antioxidant response elements (AREs) and initiate the transcription and expression of antioxidant enzyme genes [10]. Studies have shown that Nrf2 is activated by adenosine monophosphate-activated protein kinase (AMPK) [11]. Subsequently, Nrf2 activates the expression of downstream antioxidant genes, such as glutathione peroxidase (GPx), heme oxygenase 1 (HO-1), NAD (P) H : quinone oxidoreductase (NQO1), superoxide dismutase (SOD), and catalase (CAT). The expression of antioxidant genes plays a major role in antioxidant response and clear the excess ROS produced in the body, thereby reducing the myocardial damage caused by heart disease [12].

Shikonin (SKN) is one of the effective ingredients of Zicao, a traditional Chinese herbal medicine made from the dried root of *Lithospermum erythrorhizon*. It has a wide range of pharmacological effects, such as antitumor effects [13], antibacterial effects [14, 15], antivirus effects [16], antithrombosis effects [17], liver protection, and wound healing effects. Andújar et al. [18] found that in the inflammatory model of mouse ear edema induced by TPA (12-O-tetrade-canoylphorbol-13-acetate) and mouse paw edema induced by subcutaneous injection of carrageenan into the plantar foot, SKN significantly inhibits edema and exerts anti-inflammatory effects that might be related to IkB-*α* and NF-*κ*B [19]. Some studies have shown that SKN decreases the release of TNF-*α* mediated by lipopolysaccharide (LPS) in rat primary macrophage cultures by reducing the activity of the proteasome and preventing the transfer of p65-NF-*κ*B from the cytoplasm to the nucleus, thus exerting an anti-inflammatory effect [20]. Yang et al. [21] used isoproterenol to establish a mouse heart injury model and treated the mouse model with SKN orally. The study found that SKN inhibits myocardial inflammation and relieves the symptoms of heart failure. These results indicated that SKN exerts cardiovascular protective effects.

Some studies have shown that SKN plays a role in protecting angiogenesis in rheumatoid arthritis by downregulating the PI3K/AKT and MAPK signaling pathways [22]. Also, it exerts an antioxidant and an antiapoptotic effect to protect PC12 cells from *β*-amyloid peptide-induced cell damage [23], indicating that the cardioprotective effect of SKN might be partially achieved through anti-inflammatory and antioxidant effects. Mechanism studies demonstrated that SKN inhibits NF-*κ*B activation by upregulating the PI3K/Akt/Nrf2-dependent antioxidant effects in EA.hy926 endothelial cells, thereby inhibiting LDL-induced monocyte adhesion [24]. Therefore, we speculated that the protective effect of SKN on the heart might be related to the Nrf2 pathway. In the present study, we treated the human venous endothelial cell (HUVEC) injury model with SKN to explore its protective effect on cardiovascular endothelial cell injury and elucidate the underlying mechanisms.

## 2. Materials and Methods

### 2.1. Experimental Materials

SKN (purity >99.98%) was bought from Shanghai Yuanye Biotechnology Co., Ltd. (Shanghai, China, Cat No. R18D9F77950). Ox-LDL was purchased from Peking Union-Biology Co., Ltd. (Beijing, China). HUVECs were purchased from ScienCell Inc. (Carlsbad, CA, USA). DMEM (31600) and trypsin (T8150) were obtained from Beijing Solarbio Science and Technology Co., Ltd. MTT (KGA311) and Annexin V-FITC/PI apoptosis kits (KGA106) were purchased from Jiangsu KeyGEN BioTECH Corp., Ltd. (Nanjing, China). DCFH-DA (D6470) was procured from Beijing Solarbio Science and Technology Co., Ltd. SOD kit (A001-3) was purchased from Nanjing Jiancheng Bioengineering Institute (Nanjing, China). Nuclear/cytoplasmic protein extraction kit (AMJ-KT0006) was obtained from AmyJet Scientific Co., Ltd. (Wuhan, China). RIPA cell lysate (P0013E), PMSF (ST506), BCA protein content determination reagents (P0012), 10% (P0690), and 12% SDS-PAGE gel superquick preparation kits (P0692) were purchased from Beyotime Biotechnology Institute (Shanghai, China). Anti-TNF-*α* (ab6671), anti-IL-1*β* (ab234437), anti-IL-6 (ab208113), anti-VCAM1 (ab115135), anti-ICAM1 (ab171123), anti-E-selectin (ab18981), anti-AMPK (ab32047), anti-p-AMPK (ab133448), anti-Nrf2 (ab137550), anti-HO-1 (ab137749), and anti-GAPDH (ab9485) primary antibodies were purchased from Abcam (Cambridge, UK). Rabbit anti-goat secondary antibody (ZB-2306) was obtained from Zhongshan Golden Bridge Biotechnology (Beijing, China). Immobilon Western Chemiluminescent HRP Substrate (WBKLS0500) was obtained from Merck Millipore (MA, USA). siRNAs of si-Nrf2 and si-HO-1 were obtained from OBiO Technology Corp., Ltd. (Shanghai, China).

### 2.2. Cell Culture and Drug Administration

HUVECs were seeded at a density of 2 × 10^5^ cells/well in six-well plates and incubated overnight. When the cell confluency reached 80–90%, 0.25% trypsin was used to digest and passage the cells. For the cells in the drug-treated group, HUVECs were pretreated with SKN for 2 h before subsequent procedures [25].

### 2.3. siRNA Transfection

Lipofectamine 2000 was used to transfect the siRNAs of Nrf2 and HO-1 into HUVECs, respectively. After 6 h, the medium was changed, and after 24 h, the cells were treated with SKN for 2 h and then with 100 *μ*g/mL ox-LDL for 24 h [26].

### 2.4. Groups

The cells were divided into six groups. (1) Control group: the cells were cultured without treatment. (2) ox-LDL group: the cells were treated with 100 *μ*g/mL ox-LDL for 24 h. (3) SKN + ox-LDL group: the cells were treated with 1 *μ*mol/L SKN 2 h before the treatment with 100 *μ*g/mL ox-LDL for 24 h. (4) SKN + ox-LDL + compound C group: the cells were treated with 5 mmol/L compound C for 2 h, 1 *μ*mol/L SKN for 2 h, and then 100 *μ*g/mL ox-LDL for 24 h. (5) SKN + ox-LDL + si-Nrf2 group: si-Nrf2 was transfected for 24 h, followed by treatment with 1 *μ*mol/L SKN for 2 h and then 100 *μ*g/mL ox-LDL treatment for 24 h. (6) SKN + ox-LDL + si-HO-1 group: si-HO-1 was transfected for 24 h, followed by 1 *μ*mol/L SKN for 2 h and 100 *μ*g/mL ox-LDL for 24 h.

### 2.5. MTT Assay

An equivalent of 2 × 10^5^ cells/well was inoculated in a 96-well plate. Four replicates of each group were set up for the corresponding treatments, 10 *μ*L of MTT (0.5 mg/mL) was added to each well, and the cells were incubated for 4 h before the medium was discarded. Then, 150 *μ*L dimethyl sulfoxide was added to each well to stop the reaction, and the absorbance was measured at 540 nm (A540) on the microplate reader [27].

### 2.6. Detection of Apoptosis by Flow Cytometry

The cells were seeded at a density of 2 × 10^5^ per well in a six-well plate, and four replicates were set for each group, and the corresponding treatments were performed, respectively. Then, the cells were digested with trypsin (without EDTA), washed with phosphate-buffered saline (PBS), followed by centrifugation. The supernatant was discarded, and the cells were resuspended in 500 *μ*L binding buffer. Then, 10 *μ*L Annexin V-FITC and 5 *μ*L PI were added to the cells for 15 min in the dark at room temperature, and cell apoptosis was detected by flow cytometry [28].

### 2.7. Determination of ROS

The cells were seeded in a six-well plate at a density of 2 × 10^5^ cells/well. For each treatment, four replicates were set. Then, 10 *μ*mol/L DCFH-DA was added to each well and incubated in the dark for 30 min. The ROS level of each group was analyzed by flow cytometry [29].

### 2.8. SOD Content Determination

The SOD content was determined according to the manufacturer's instructions.

### 2.9. Protein Extraction

The cells were seeded in a 24-well plate at a density of 2 × 10^5^ cells/well, with four replicate wells in each group, and the corresponding treatments were performed, respectively. A mixture of RIPA lysis buffer and PMSF (100 : 1) was added to each well to lyse the cells for 15 min, and the supernatant was collected by centrifugation to extract total protein. The nucleoprotein was extracted according to the kit instructions, and the BCA protein kit was used for protein quantification.

### 2.10. Western Blot to Detect Protein Levels

An equivalent of 40 *μ*g protein sample was subjected to SDS-PAGE and transferred to nitrocellulose membranes in Tris-glycine buffer at 100 V for 1 h. The membranes were blocked with 5% skimmed milk powder at room temperature for 1 h, probed with the primary antibody (1 : 1000) at 4°C, and then added the secondary antibody (1 : 5000) at room temperature for 1 h. The immunoreactive signals were detected using ECL reagent, and AI 600 multifunction imaging system was used for imaging, and the relative intensities of protein bands were quantified using Image J software [30].

### 2.11. Statistical Analysis

GraphPad Prism5.0 software was used for data analysis. The data were expressed as mean ± standard deviation (SD), and the differences between the groups were analyzed using one-way analysis of variance (ANOVA), followed by Dunnett's or Tukey's post-hoc tests. *P* < 0.05 indicated a statistically significant difference.

## 3. Results

### 3.1. SKN Improves the Viability of Endothelial Cells Induced by ox-LDL

**Figure 1:**
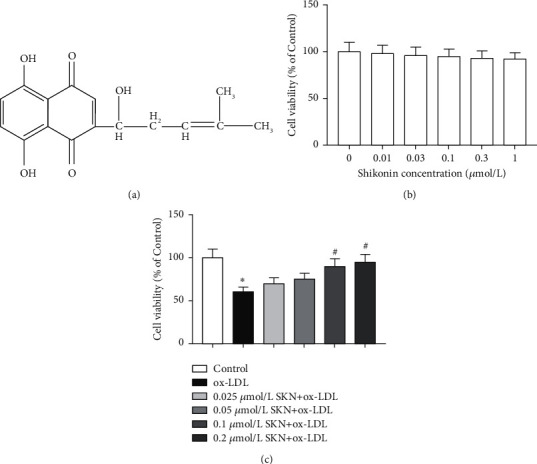
Effects of SKN on ox-LDL-treated HUVECs. (a) Chemical structure of SKN. (b) Effects of SKN on HUVECs. (c) Effects of SKN on cell viability in ox-LDL-treated HUVECs. Data are expressed as mean ± SD, *n* = 3. ^*∗*^*P* < 0.05 vs. the control group. ^#^*P* < 0.05 vs. the ox-LDL group.

The chemical structure of SKN is shown in Figure 1(a). As shown in Figure 1(b), the MTT method was used to detect the effect of SKN on the viability of endothelial cells. Compared to the control group, the viability of cells after pretreatment with different concentrations of SKN did not change significantly, indicating that SKN did not exert any toxic effect on HUVECs. In Figure 1(c), the results showed that compared to the control group, ox-LDL treatment significantly decreased the cell viability of HUVECs, while it increased in an SKN concentration-dependent manner. When the shikonin concentration was >0.1 *μ*mol/L, the cell viability increased significantly.

### 3.2. SKN Reduces ox-LDL-Induced Endothelial Cell Apoptosis

**Figure 2:**
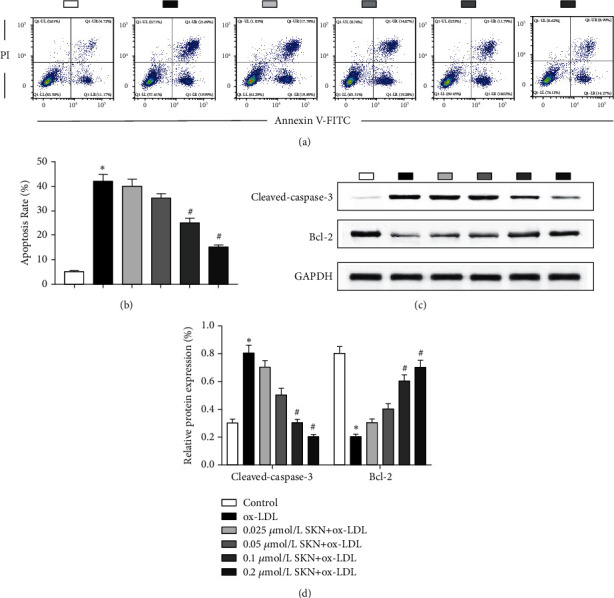
Effects of SKN on cell apoptosis in ox-LDL-treated HUVECs. (a-b) Effects of SKN on apoptosis rate in ox-LDL-treated HUVECs. (c-d) Effects of SKN on the expression level of apoptosis-related proteins, cleaved caspase-3, and Bcl-2. Data are expressed as mean ± SD, *n* = 3. ^*∗*^*P* < 0.05 vs. the control group. ^#^*P* < 0.05 vs. the ox-LDL group.

Flow cytometry was used to detect cell apoptosis (Figures 2(a) and 2(b)). Compared to the control group, ox-LDL significantly increased the level of cell apoptosis. Compared to the ox-LDL group, SKN pretreatment decreases the apoptosis rate in a concentration-dependent manner. When the concentration is > 0.1 *μ*mol/L, the difference is statistically significant (*P* < 0.05). The expression of apoptosis-related proteins was further detected by Western blot (Figures 2(c) and 2(d)). Compared to the control group, the expression of cleaved caspase-3 protein in the ox-LDL group was significantly increased, while that of Bcl-2 was significantly reduced. SKN pretreatment downregulated the cleaved caspase-3 protein levels and upregulated Bcl-2 protein levels in a concentration-dependent manner. Follow-up experiments chose 0.1 *μ*mol/L as the treatment concentration of SKN.

### 3.3. SKN Downregulates the Expression of Inflammatory Factors Induced by ox-LDL

**Figure 3:**
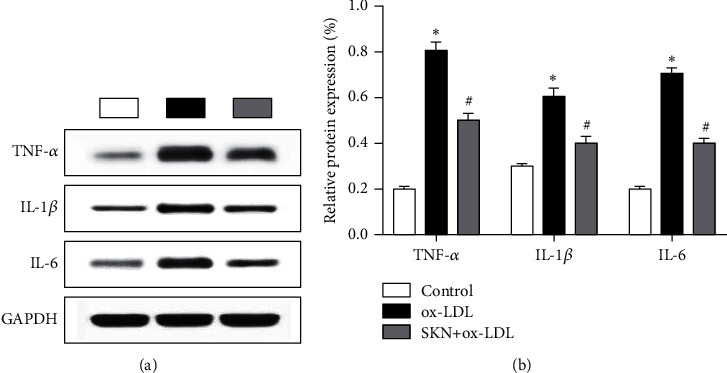
Effects of SKN on the expression of TNF-*α*, IL-1*β*, and IL-6 in ox-LDL-treated HUVECs. (a) Results of Western blot. (b) Analysis of relative protein expression. Data are expressed as mean ± SD, *n* = 3. ^*∗*^*P* < 0.05 vs. the control group. ^#^*P* < 0.05 vs. the ox-LDL group.

Western blot was used to detect the levels of TNF-*α*, IL-1*β*, and IL-6 levels, as shown in Figures 3(a) and 3(b). Compared to the control group, ox-LDL can significantly upregulate the levels of TNF-*α*, IL-1*β*, and IL-6 proteins and downregulate the expression levels of ox-LDL-induced TNF-*α*, IL-1*β*, and IL-6 proteins.

### 3.4. SKN Downregulates the Expression of ox-LDL-Induced Adhesion Molecules

**Figure 4:**
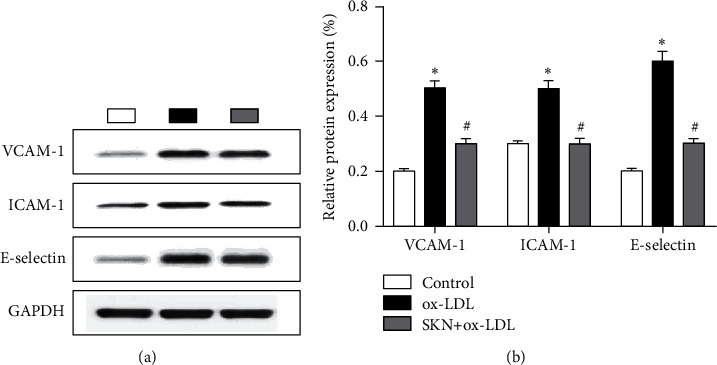
Effects of SKN on the expression of VCAM1, ICAM1, and E-selectin in ox-LDL-treated HUVECs. (a) Results of Western blot. (b) Analysis of protein expression. Data are expressed as mean ± SD, *n* = 3. ^*∗*^*P* < 0.05 vs. the control group. ^#^*P* < 0.05 vs. the ox-LDL group.

Western blot was used to detect the protein levels of vascular cell adhesion molecule-1 (VCAM1), intercellular cell adhesion molecule-1 (ICAM1), and E-selectin, as shown in Figure 4. The results showed that compared to the control group, ox-LDL significantly upregulates the protein level of VCAM1, ICAM1, and E-selectin, while SKN pretreatment significantly downregulates the levels of VCAM1, ICAM1, and E-selectin proteins.

### 3.5. SKN Inhibits ox-LDL-Induced Oxidative Stress

**Figure 5:**
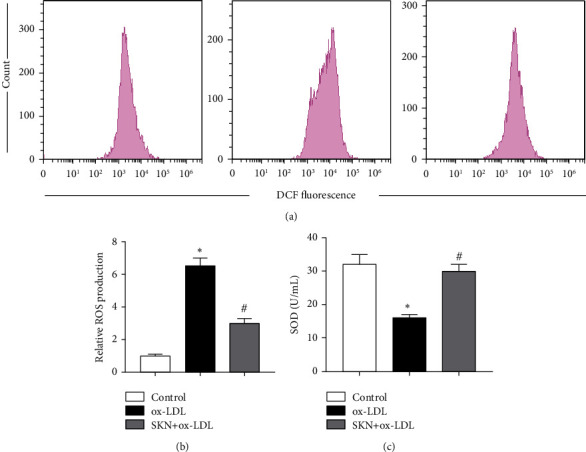
Effects of SKN on oxidative stress in ox-LDL-treated HUVECs. (a-b) Relative ROS production of cells in the three groups. (c) SOD activity of cells in the three groups. Data are expressed as mean ± SD, *n* = 3. ^*∗*^*P* < 0.05 vs. the control group. ^#^*P* < 0.05 vs. the ox-LDL group.

As shown in Figure 5, compared to the control group, ox-LDL significantly upregulates the production of ROS and reduces SOD activity. Compared to the ox-LDL group, SKN pretreatment significantly decreases the generation of ROS and increases the SOD activity induced by ox-LDL.

### 3.6. SKN Inhibits Oxidative Stress Damage by Activating the AMPK-Nrf2-HO-1 Pathway

**Figure 6:**
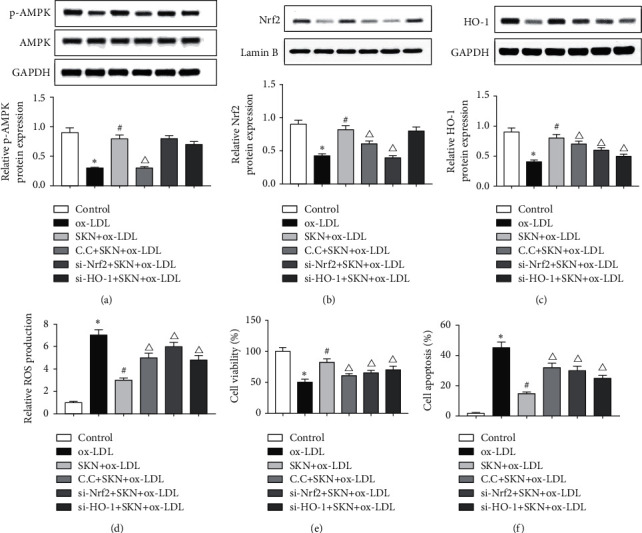
Effects of SKN on AMPK-Nrf2-HO-1 signaling in ox-LDL-treated HUVECs. (a) Results of Western blot and analysis of AMPK and p-AMPK in ox-LDL-treated HUVECs. (b) Results of Western blot and analysis of Nrf2 in ox-LDL-treated HUVECs. (c) Results of Western blot and analysis of HO-1 in ox-LDL-treated HUVECs. (d) Relative ROS production of cells in different groups. (e) Cell viability in different groups. (f) Cell apoptosis rate in ox-LDL-treated HUVECs. Data are expressed as mean ± SD, *n* = 3. ^*∗*^*P* < 0.05 vs. the control group. ^#^*P* < 0.05 vs. the ox-LDL group. Δ*P* < 0.05 vs. the SKN + ox-LDL group.

As shown in Figures 6(a)–6(c), the levels of p-AMPK, AMPK, Nrf2, and HO-1 proteins were detected in each group of cells by Western blot. The results showed that compared to the control group, the expression of p-AMPK, Nrf2, and HO-1 proteins decreased significantly in the ox-LDL group, while SKN treatment significantly increased the protein levels.

In order to determine the correlation between SKN and AMPK-Nrf2-HO-1 signaling pathway, the inhibitor of AMPK compound C, si-Nrf2, and si-HO-1 inhibited the expression of this pathway. The results showed that compared to the SKN + ox-LDL group, the expression of p-AMPK, Nrf2, and HO-1 proteins in the CC + SKN + ox-LDL group was significantly downregulated, while that of Nrf2 in the si-Nrf2+SKN + ox-LDL group was significantly downregulated and the expression level of p-AMPK did not change significantly. However, the expression of HO-1 was significantly downregulated, but that of p-AMPK was not altered significantly. In addition, compared to the SKN + ox-LDL group, compound C, si-Nrf2, and si-HO-1 treatment groups had significantly increased ROS production (Figure 6(d)). MTT and flow cytometry results showed that compared to the SKN + ox-LDL group, the cell viability of compound C, si-Nrf2, and si-HO-1 treatment groups decreased significantly, while the apoptosis rate increased significantly (Figures 6(e) and 6(f)). The above results showed that SKN reduces ox-LDL-induced oxidative stress and other damages by activating the AMPK-Nrf2-HO-1 pathway.

## 4. Discussion

AS is a common cardiovascular disease, caused by many factors, such as hypertension, hyperglycemia, hyperlipoidemia, smoking, and drinking, which seriously threatens human health. The pathogenesis of AS has not yet been clarified, but it is speculated that vascular endothelial injury is the main pathological change of the occurrence and development of AS and constitutes a complex process including apoptosis, inflammation, and oxidative stress [31].

Under normal physiological conditions, the proliferation and apoptosis of endothelial cells maintain a dynamic balance in blood vessels. Previous studies have shown extensive endothelial cell apoptosis in AS plaques, and excessive endothelial cell apoptosis is the initial factor of endothelial dysfunction [3]. In this study, for the first time, we measured the cell viability by MTT assay and found that ox-LDL significantly decreased the cell viability of HUVECs. SKN increased the cell viability of HUVECs in a concentration-dependent manner, indicating that shikonin alleviates ox-LDL-induced cell damage. Flow cytometry was used to detect the level of apoptosis, and the results showed that ox-LDL significantly increases the apoptosis rate of HUVECs, and SKN decreases the ox-LDL-induced apoptosis in a concentration-dependent manner. In this experiment, we further detected the level of apoptosis-related proteins by Western blot and found that SKN downregulates the xo-LDL-induced cleaved caspase-3 level in a concentration-dependent manner and upregulates Bcl-2 protein level. These results indicated that SKN protects HUVECs from ox-LDL-induced damage in a concentration-dependent manner.

The inflammatory response of the vascular endothelium plays a major role in the occurrence and development of AS. The rolling, adhesion, migration, and accumulation of white blood cells are the features of the inflammatory response in the vascular endothelium [32]. Under the stimulation of external environmental factors, endothelial cells can be stimulated to produce a variety of inflammatory factors and adhesion molecules, accelerate the adhesion of white blood cells to endothelial cells, and promote the occurrence of inflammation. Jiang et al. [33] demonstrated that IL-6, TNF-*α*, VCAM1, ICAM1, and E-selectin levels increased significantly when the HUVECs were treated with ox-LDL. Some studies showed that SKN has an effect against AS by reducing the expression of inflammatory factors and adhesion factors [34]. The current results showed that ox-LDL significantly upregulated the expression of TNF-*α*, IL-1*β*, IL-6, VCAM1, ICAM1, and E-selectin in endothelial cells, while SKN treatment significantly downregulated the expression of these proteins mentioned above, which was consistent with those of previous studies. These findings indicated that SKN can protect HUVECs from the inflammatory response induced by ox-LDL.

Reportedly, abnormal apoptosis and inflammatory response are closely related to oxidative stress that occurs when excessive ROS is generated, and the biological activity exceeds the body's antioxidant capacity. In various cardiovascular diseases, such as metabolic syndrome and dyslipidemia, the level of ROS increased significantly [35, 36]. SOD is vital antioxidase that protects the body from oxidative damage [37]. Our findings indicated that ox-LDL significantly upregulates the ROS production and downregulates the SOD expression, while SKN significantly downregulates ox-LDL-induced ROS production and upregulates SOD expression. This result indicated that SKN protects HUVECs from co-LDL-induced oxidative stress. Nrf2 is a transcription factor that regulates cellular oxidative stress response and is also a central regulator of intracellular redox homeostasis [38]. The stimulation and regulation of the constitutive and inducible expression of the antioxidant protein, Nrf2, protects the cell damage caused by ROS and electrophiles and maintains the cells in a stable state and the body's redox homeostasis [39]. Nrf2 also regulates the expression of various antioxidant enzymes, such as HO-1 [40, 41], which has cytoprotective effects [42, 43]. Therefore, the activation of the Nrf2/HO-1 signaling pathway protects the cells from oxidative damage.

Adenosine 5′-monophosphate (AMP)-activated protein kinase (AMPK) is a serine/threonine protein kinase. In addition, the activation of AMPK inhibits abnormal inflammation and oxidative stress [44]. In the present study, Western blot results showed that ox-LDL significantly reduced the levels of p-AMPK, Nrf2, and HO-1 levels, while SKN pretreatment significantly increased the expression of p-AMPK, Nrf2, and HO-1 proteins and reduced the production of ROS by increasing the level of Nrf2. Some studies showed that SKN inhibits ROS production through PI3K/Akt/Nrf2-dependent antioxidant enzyme expression [24]. Recent experiments found that AMPK is activated by oxidative stress and then regulates the antioxidant substances in the body (hypoxic activation of AMPK is dependent on mitochondrial ROS but independent of an increase in AMP/ATP ratio) [45]. In order to further determine the correlation between the AMPK-Nrf2-HO-1 pathway and the protective effect of SKN, we used AMPK inhibitor compound C, si-Nrf2, and si-HO-1 to inhibit the expression of the signaling pathway proteins. The results showed that compared to compound C, si-Nrf2, and si-HO-1 groups, SKN reduces ROS production. MTT and flow cytometry showed that compared to compound C, si-Nrf2, and si-HO-1 groups, the cell viability of SKN + ox-LDL treatment was significantly increased, while the apoptosis rate was decreased, indicating that SKN can reduce the oxidative stress and cell injury induced by ox-LDL via the activated AMPK-Nrf2-HO-1 signaling pathway.

In summary, SKN suppressed ox-LDL-induced ROS production, improved cell viability, and cell apoptosis via the AMPK/Nrf2/HO-1 pathway.

## Data Availability

The datasets used and/or analyzed during the current study are available from the corresponding author upon request.
